# Towards atomic resolution in sodium titanate nanotubes using near-edge X-ray-absorption fine-structure spectromicroscopy combined with multichannel multiple-scattering calculations

**DOI:** 10.3762/bjnano.3.88

**Published:** 2012-11-23

**Authors:** Carla Bittencourt, Peter Krüger, Maureen J Lagos, Xiaoxing Ke, Gustaaf Van Tendeloo, Chris Ewels, Polona Umek, Peter Guttmann

**Affiliations:** 1 ChIPS, University of Mons, B-7000, Mons, Belgium; 2 ICB, UMR 6303 CNRS-Université de Bourgogne, F-21078 Dijon, France; 3 EMAT, University of Antwerp, B-2020, Antwerp, Belgium; 4 Institut des Matériaux de Nantes (IMN), Université de Nantes, CNRS, Nantes, France; 5 Jožef Stefan Institute, Jamova cesta 39, 1000 Ljubljana, Slovenia; 6 Center of Excellence NAMASTE, Jamova cesta 39, 10000 Ljubljana, Slovenia; 7 Helmholtz-Zentrum Berlin für Materialien und Energie GmbH, Institute for Soft Matter and Functional Materials, Albert-Einstein-Str. 15, 12489 Berlin, Germany

**Keywords:** multichannel multiple scattering, nanotubes, NEXAFS, sodium titanates

## Abstract

Recent advances in near-edge X-ray-absorption fine-structure spectroscopy coupled with transmission X-ray microscopy (NEXAFS–TXM) allow large-area mapping investigations of individual nano-objects with spectral resolution up to *E*/Δ*E* = 10^4^ and spatial resolution approaching 10 nm. While the state-of-the-art spatial resolution of X-ray microscopy is limited by nanostructuring process constrains of the objective zone plate, we show here that it is possible to overcome this through close coupling with high-level theoretical modelling. Taking the example of isolated bundles of hydrothermally prepared sodium titanate nanotubes ((Na,H)TiNTs) we are able to unravel the complex nanoscale structure from the NEXAFS–TXM data using multichannel multiple-scattering calculations, to the extent of being able to associate specific spectral features in the O K-edge and Ti L-edge with oxygen atoms in distinct sites within the lattice. These can even be distinguished from the contribution of different hydroxyl groups to the electronic structure of the (Na,H)TiNTs.

## Introduction

Transmission X-ray microscopy (TXM) is a popular microscopy technique used in biology [1–3]. Recently, we have extended the range of its applications to the spectroscopic characterization of nanoscale materials by combining it with near-edge X-ray-absorption fine-structure spectroscopy (NEXAFS–TXM) [4]. The nanoscale spatial resolution of the NEXAFS–TXM allows the exclusion of impurity regions and thus the presence of signals related to impurities in the absorption spectrum of the nanostructures. State of the art TXMs allow a spatial resolution of 11 nm [5–7]. However, we show in the current study that the combination of NEXAFS–TXM with high-level theoretical modelling allows us to move beyond this spatial-resolution limit and extract more spatially refined information [8]. Indeed this combination of theory and spectroscopy paves the way towards atomic-scale resolution in similar techniques with lower spectral resolution [9–10].

We investigate here the electronic structure of sodium titanate nanotubes ((Na,H)TiNTs) by means of near-edge X-ray-absorption fine-structure spectroscopy (NEXAFS) coupled with first-principles NEXAFS calculations (density functional for the O K-edge and multichannel multiple scattering (MCMS) method for the Ti L_2,3_-edge spectra) [8,11–12]. The susceptibility of both O K-edge and Ti L-edge features to the local bonding environment in TiO_2_-based materials makes NEXAFS ideal for providing diagnostic information about the crystal structures and oxidation states. Here, the electronic structure of the nanotubes is discussed in terms of the ligand field splitting of the Ti ions and the connectivity of the TiO_6_ octahedral network. Among the different structures proposed for these nanotubes [13–17], it is currently accepted that the structure of the layered titanate H_2_Ti*_n_*O_2_*_n_*_+1_ better describes the (Na,H)TiNTs [17–18].

Potential applications in lithium-ion batteries, catalyst supports, photocatalysts, and dye-synthesized solar cells have effectively resulted in an increasing interest in titanate nanostuctures [19–29]. All these applications require a deep understanding of the electronic structure of the material. In addition to spatial resolution, the NEXAFS–TXM offers higher-energy resolution and lower-damage yield when compared to other advanced spectromicroscopy techniques, such as electron energy loss spectroscopy (EELS) performed in aberration-corrected transmission electron microscopes operated at low electron acceleration voltages [30]. Alkali titanate nanostructures are very sensitive to electron-beam irradiation, which has prevented detailed studies at high energetic resolution by electron spectroscopy. Using NEXAFS–TXM has allowed us to probe our samples at 0.1 eV energy resolution while in recent reports [18,31] based on EELS the energy resolution for analysing similar samples was limited to 0.5 eV.

## Results and Discussion

**Figure 1:**
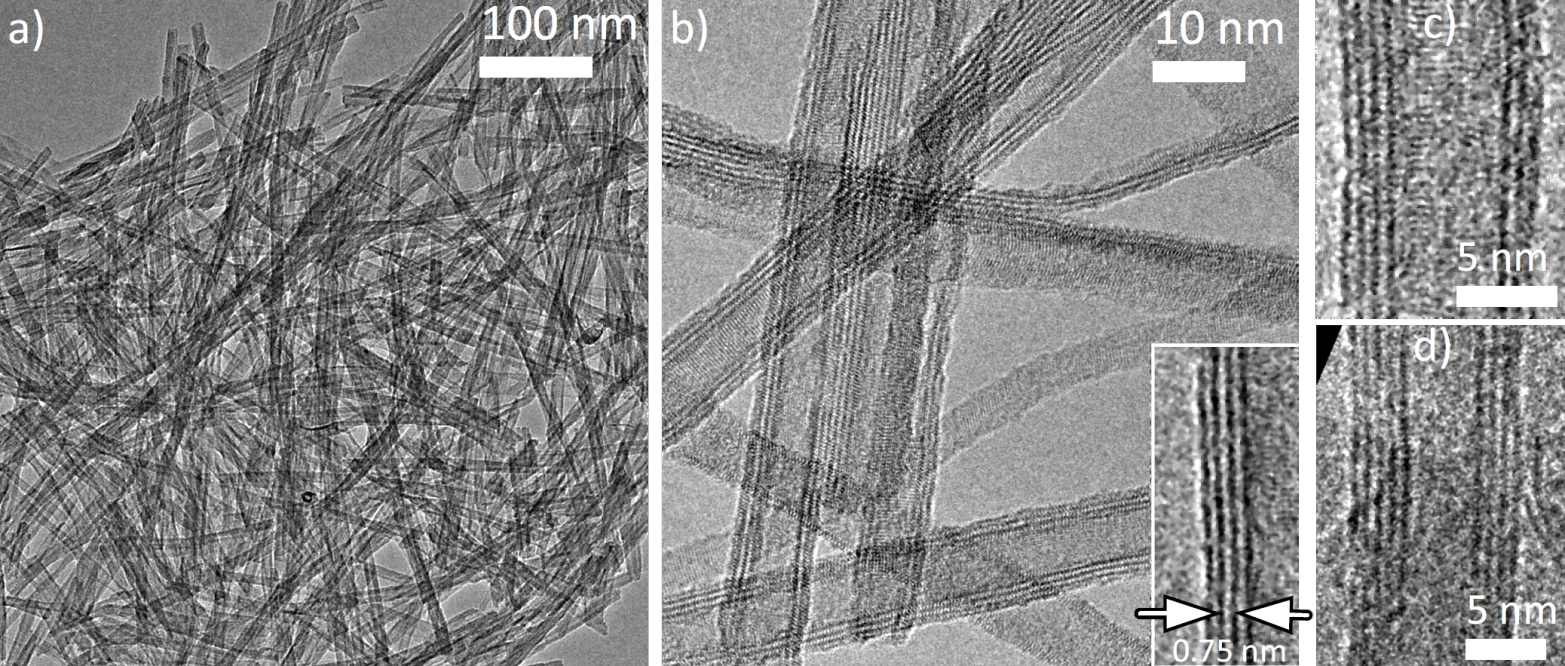
TEM image of sodium titanate nanotubes (a) typical region used to record the NEXAFS–TXM data. (b) HRTEM image revealing the hollow core of these nanostructures (inset shows the interlayer spacing). (c–d) HRTEM images showing typical image contrast pattern associated with the scroll-like morphology. Note the number of layers in opposing tube walls is different in each case.

Figure 1a shows a typical TEM image of the sodium titanate nanostructures. Figure 1b shows a high-resolution TEM image of several long structures, showing parallel dark contrast, typical for tubular morphology (see Supporting Information File 1). The nanotube edges consist of several layers spaced by 0.75 nm (see Figure 1 insert). Typical high-resolution TEM images (Figure 1c and Figure 1d) show that the tubes have a different number of layers in each edge (for instance Figure 1c has four layers on the left, three on the right), suggesting that these are very likely hollow nanoscrolls, consistent with previous observations [11,32]. Characteristic nanoscrolls have walls consisting of 2–6 layers, the outer diameters of the synthesized nanotubes are between 8 and 12 nm, while the inner diameters are found in the range between 4 and 7 nm. They display high aspect ratio with lengths of a few hundred nanometers.

**Figure 2:**
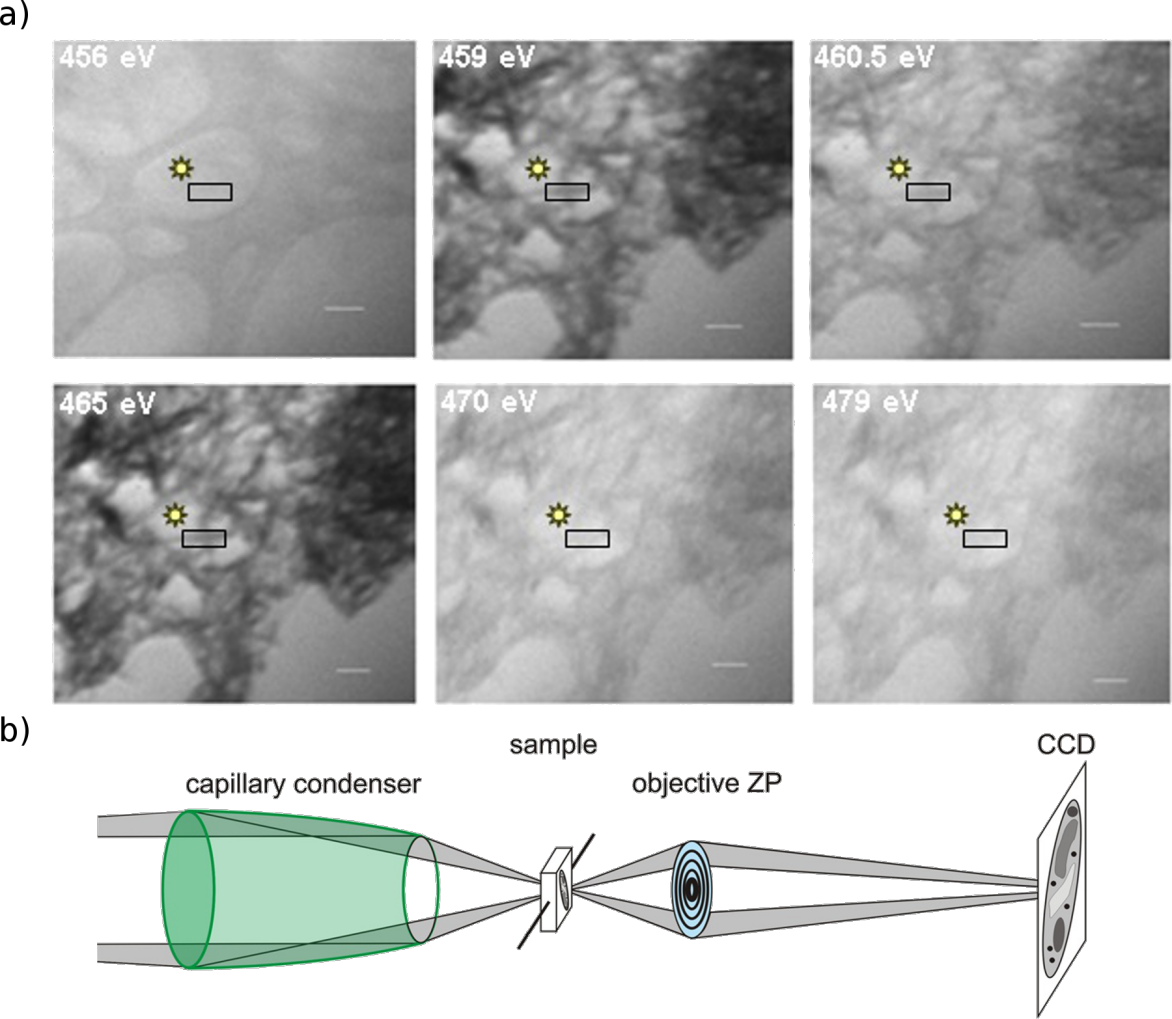
(a) X-ray images at different photon energies *E* from an image stack recorded on the (Na,H)TiNTs at the U41 beamline by using the TXM. The NEXAFS–TXM (Ti L-edge (see Figure 3a) and O K-edge (see Figure 5)) spectra were recorded in the area delimited by the rectangle. The spot indicates the point at which the *I*_0_ was recorded. (b) Setup of the NEXAFS–TXM measurements.

The NEXAFS spectra were recorded in the transmission mode with a zone plate objective with an outermost zone width of 40 nm. This allows the study of selected areas of the samples and reduces the uncertainties due to the presence of impurities. Figure 2 shows a few X-ray images of the image stack used to record the NEXAFS spectra. For absorption spectroscopy it is necessary to measure two spectra: one spectrum *I*(*E*) of transmission through the specimen and another of the incident flux *I*_0_(*E*). The spectrum is obtained as an optical density OD(*E*) = −log [*I*(*E*)/*I*_0_(*E*)] [33]. A region containing a bundle with a few (Na,H)TiNTs was used to record the *I*(*E*) spectrum, and the *I*_0_(*E*) was recorded in a bare region of the sample support close to the sample region (Figure 2). During the measurements the system was kept in focus, and the alignment of the images was performed by using a cross-correlation method from the IMOD tomography package [34].

**Figure 3:**
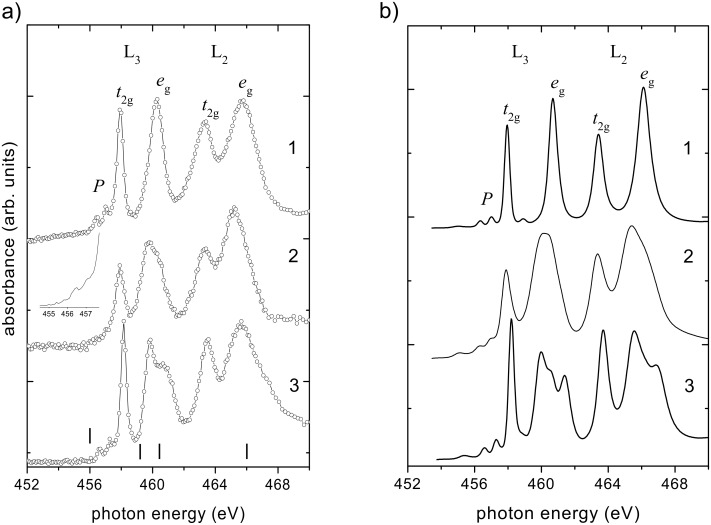
NEXAFS spectra at the Ti L-edge recorded on (1) SrTiO_3_, (2) (Na,H)TiNTs and (3) anatase. The vertical lines indicate the photon energy of the first four X-ray images in Figure 2. The inset shows the pre-edge structures in the nanotube spectrum. (b) Ti L-edge spectra of (1) SrTiO_3_, (2) (Na,H)TiNTs and (3) anatase calculated with the MCMS method.

The titanium L-edge NEXAFS spectrum of the (Na,H)TiNTs shares common general features with the spectra recorded on anatase and SrTiO_3_ (Figure 3): they are composed of distinguishable peaks in the range between 455 and 470 eV corresponding to excitations of the Ti 2p states into the empty Ti 3d states [35]. Due to the hydrothermal process used to synthesize the tubes, the binding energy of Ti 2p photoemission lines are shifted slightly to lower binding energy, showing that the local environment around the Ti ions in the (Na,H)TiNTs is different from that in the anatase-type TiO_2_ (see also Supporting Information File 1, Figure S1). The absence of extra peaks or broadening of the Ti 2p photoemission lines suggest that the chemical environment of the Ti ions does not change during the hydrothermal treatment of TiO_2._

The Ti L-edge shows two groups of peaks arising from the spin-orbit splitting of the Ti 2p core level into 2p_1/2_ (L_2_-edge) and 2p_3/2_ levels (L_3_-edge), corresponding to Ti(IV) in a tetragonal structure [36]. These levels are then further split by the strong ligand field arising from the surrounding oxygen atoms. For TiO_6_ octahedra, even if distorted, the cubic component dominates the ligand field [37]. The cubic field splits the Ti 3d band into two sublevels with t_2g_ and e_g_ symmetry [38]. In the following we shall label the Ti 3d states with these approximate characters for simplicity, even when the exact point symmetry of the Ti site is much lower than O_h_, and so the degeneracy within the t_2g_ and e_g_ groups is lifted, as in the case of the nanotubes. The sharp L_3_*–*t_2g_ feature reflects the weak interaction between the O 2p orbitals forming directional “π-type” bonds and the t_2g_ orbitals (d*_xy_*, d*_xz_* and d*_yz_*) pointing between the oxygen neighbors. Conversely, the O 2p orbitals form strong directional bonds with the e_g_ orbitals 
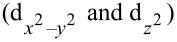
 that point directly towards the oxygen ligands and, therefore, the line-shape of the e_g_ band is highly sensitive to the local symmetry around the metal cations [39]. In Figure 3a, the structure of the L_3_*–*e_g_ transition seems to be characteristic of a tetragonal system [36,40–41]. At energies below the L_3_-edge, the low-intensity peaks observed in all samples were reported to arise from the spin-forbidden p_3/2_→d_3/2_ transition [38] and are related to particle–hole Coulomb coupling [8].

**Figure 4:**
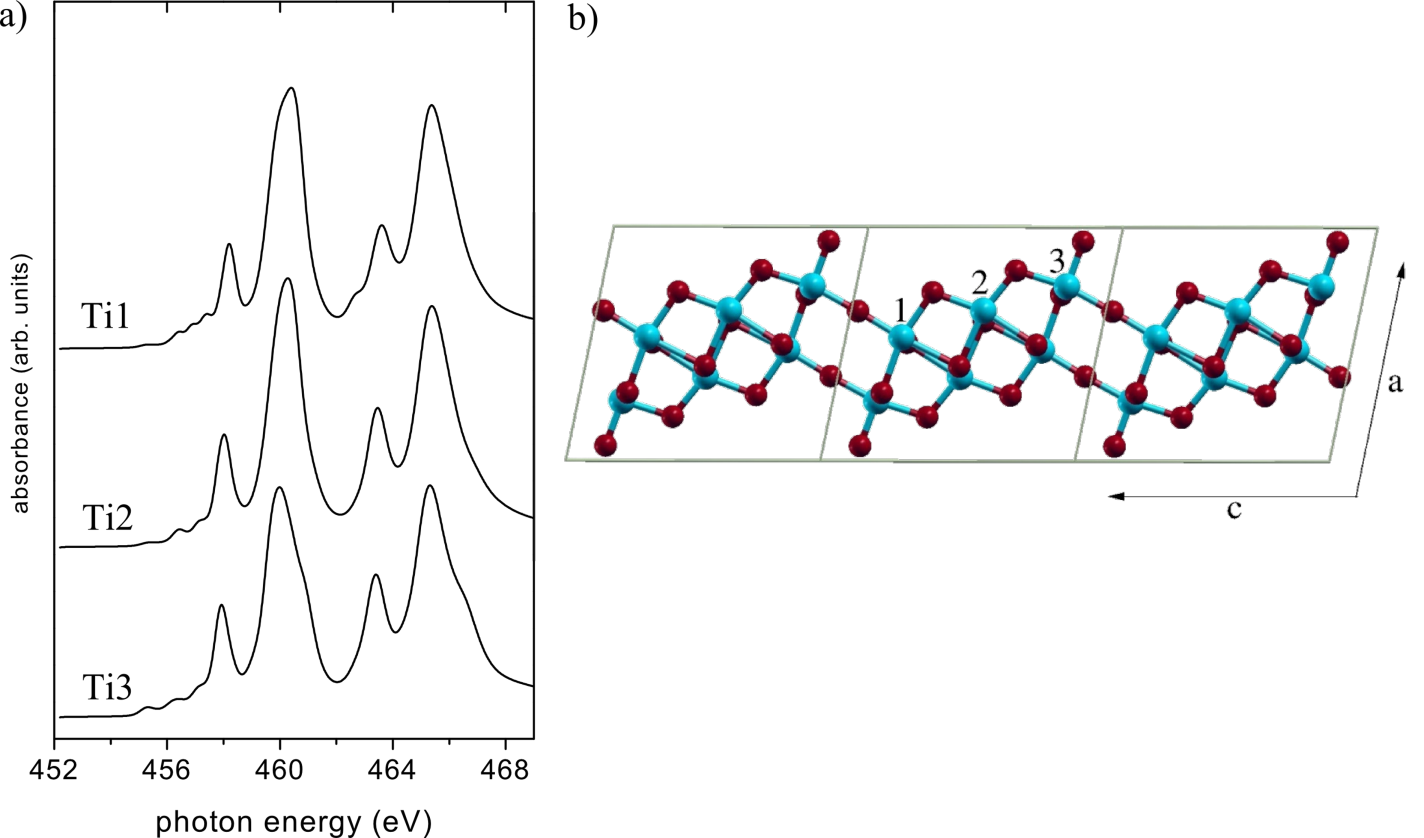
(a) Calculated NEXAFS spectra. Ti atoms at different sites of the (Na,H)TiNTs structure (Figure 4b). (b) Ball-and-stick model of one Ti_3_O_7_ sheet of the generally accepted structure of (Na,H)TiNTs [16]. Ti atoms in blue with labels 1–3 for the three nonequivalent sites, and O atoms in red. Three unit cells are shown in side view (along −b).

The most prominent difference between the spectra of the studied oxides is the value of the energy splitting of the fine structure in the L_3_*–*e_g_ band. From Figure 3a, we can see that this value is 0, 0.44 and 0.82 eV, respectively, for SrTiO_3_, (Na,H)TiNTs and anatase. Krüger showed that the L_3_*–*e_g_ peak splitting in TiO_2_ is a band-structure effect, which mainly reflects the connectivity of the TiO_6_ octahedra rather than local distortions of the individual octahedra [8]. In the SrTiO_3_ structure all octahedra are connected by their corners such that the oxygen atoms have coordination 2. In anatase the oxygen atoms have coordination 3 and connect one corner- and two edge-sharing octahedra. Considering the value of the energy split of the L_3_*–*e_g_ band evaluated from the NEXAFS spectra, half way between SrTiO_3_ and anatase, we can postulate a structure having oxygen atoms with an average coordination number of 2.5 for our scrolls. The generally accepted model for the structure of the (Na,H)TiNTs can be described as weakly bent sheets made of two layers of Ti*–*O_6_ octahedra, not stacked in perfect registry [17]. In this model, the different O sites have one to four O–Ti bonds with bond lengths ranging between 1.7 and 2.4 Å. The average coordination of the O sites is 2.57, in good agreement with our value. The unit cell has a Ti_6_O_14_ basis with three nonequivalent Ti sites (see Figure 4).

In order to move beyond the resolution limits imposed by the experiment, we next perform a series of MCMS calculations [8] using the structural model “H(1,3)” for H_2_Ti_3_O_7_ given in [17]. The finite-cluster multiple-scattering calculations were done for a disk of 148 atoms in a single titanate sheet. The atomic data (phase shifts and radial matrix elements) were calculated from self-consistent potentials of bulk TiO_2_ anatase [8]. We also tested potentials of bulk SrTiO_3_ and obtained only negligible differences in spectral line shape. The calculated Ti L_2,3_ spectra of the (Na,H)TiNTs is shown in Figure 3b along with those of anatase and SrTiO_3_. The latter were, for direct comparison, recalculated here by using the same cluster size (~150 atoms) and the same atomic potentials as the (Na,H)TiNTs. All calculated spectra were rigidly shifted by −14.5 eV to match the experimental energy scale, other computational details are the same as in [8].

The agreement between experimental and calculated spectra in Figure 3 is remarkable. All major differences between the (Na,H)TiNTs spectra and the reference spectra are well reproduced in the calculation. In the (Na,H)TiNTs, the L_3_–t_2g_ lines and L_2_–t_2g_ lines are broader and considerably less intense than those in the bulk phases. For both L_3_ and L_2_, the t_2g_*–*e_g_ splitting is reduced, especially as compared to SrTiO_3_. The weak pre-edge peaks are much more broadened than in SrTiO_3_ and TiO_2_. As for the L_3_*–*e_g_ peak, the calculation gives a more rounded shape than the data, but the characteristic width (or splitting) of the peak lies between SrTiO_3_ and anatase in excellent agreement with experiment.

**Figure 5:**
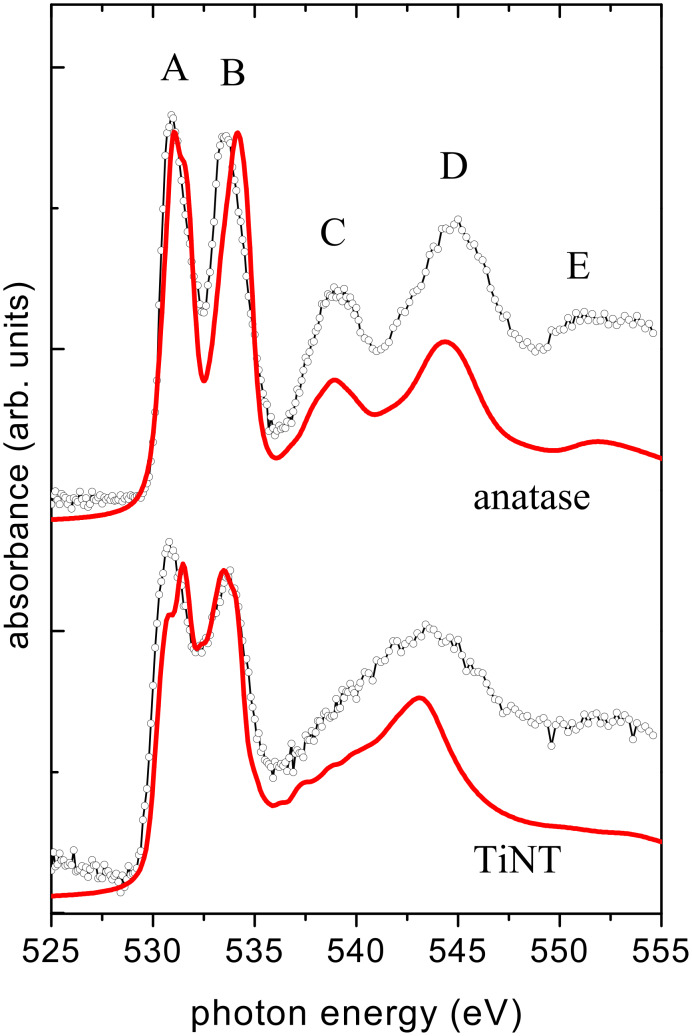
Comparison of NEXAFS spectra calculated by using density functional theory (red) and O K-edge spectra recorded on anatase and (Na,H)TiNTs (black).

Further information on the electronic state can be obtained by the analysis of the O K-edge. Figure 5 shows the O K-edge NEXAFS spectra of (Na,H)TiNTs and TiO_2_ anatase, the latter compares well with the literature [38,42]. The peaks A and B just above threshold correspond to Ti-3d–O-2p antibonding states. They are mainly of Ti-3d character and split into t_2g_ (A) and e_g_ (B) by the octahedral ligand field. The higher energy peaks (C, D and E) are due to more delocalized states that have been attributed to the hybridization of O p with Ti sp. In the (Na,H)TiNT spectrum, peaks A and B have nearly the same energy positions as in anatase, reflecting the octahedral coordination of Ti sites in both structures. The only obvious difference is that the peaks A, B are somewhat broader in the (Na,H)TiNTs. Much more pronounced changes are observed for higher energy peaks. In the (Na,H)TiNT spectrum peak C is strongly suppressed and D is shifted to lower energy such that these two peaks have merged into a single asymmetric peak. Peak E is slightly weaker than in anatase. Since the peaks C–E correspond to hybridization of O p with delocalized states, they are sensitive to the structural changes around the O sites, rather than around the Ti sites, as in the case of peaks A and B. The pronounced difference between the (Na,H)TiTNs and anatase observed in this spectral region thus indicates a very different connectivity of the TiO_6_ octahedra. However, the resolution limits of the NEXAFS–TXM approach do not allow further discrimination with the spectra based on local structure, and for this we now turn to the simulations.

In Figure 5 the O K-edge spectra are compared with density functional calculations performed in the local density approximation with the Vienna ab initio package [43–44]. As for the Ti L_23_-edge, the structural model “H(1,3)” for H_2_TiO_3_ of [23] is used for the (Na,H)TiNTs. As the core hole was found to have a negligible effect on the O K-edge NEXAFS spectra of TiO_2_, the absorption spectra can be modeled by using the O p projected ground-state density of states (DOS) [42]. The DOS was broadened with a Lorentzian of energy-dependent width, to account for finite core hole lifetime and photoelectron mean free path (see the Supporting Information File 1 for details).

The calculated spectra agree very well with the experimental data. In anatase, all peaks A–E are reproduced both in position and relative intensity. Importantly, all differences observed in the (Na,H)TiNT spectrum are also reproduced in the calculation.

**Figure 6:**
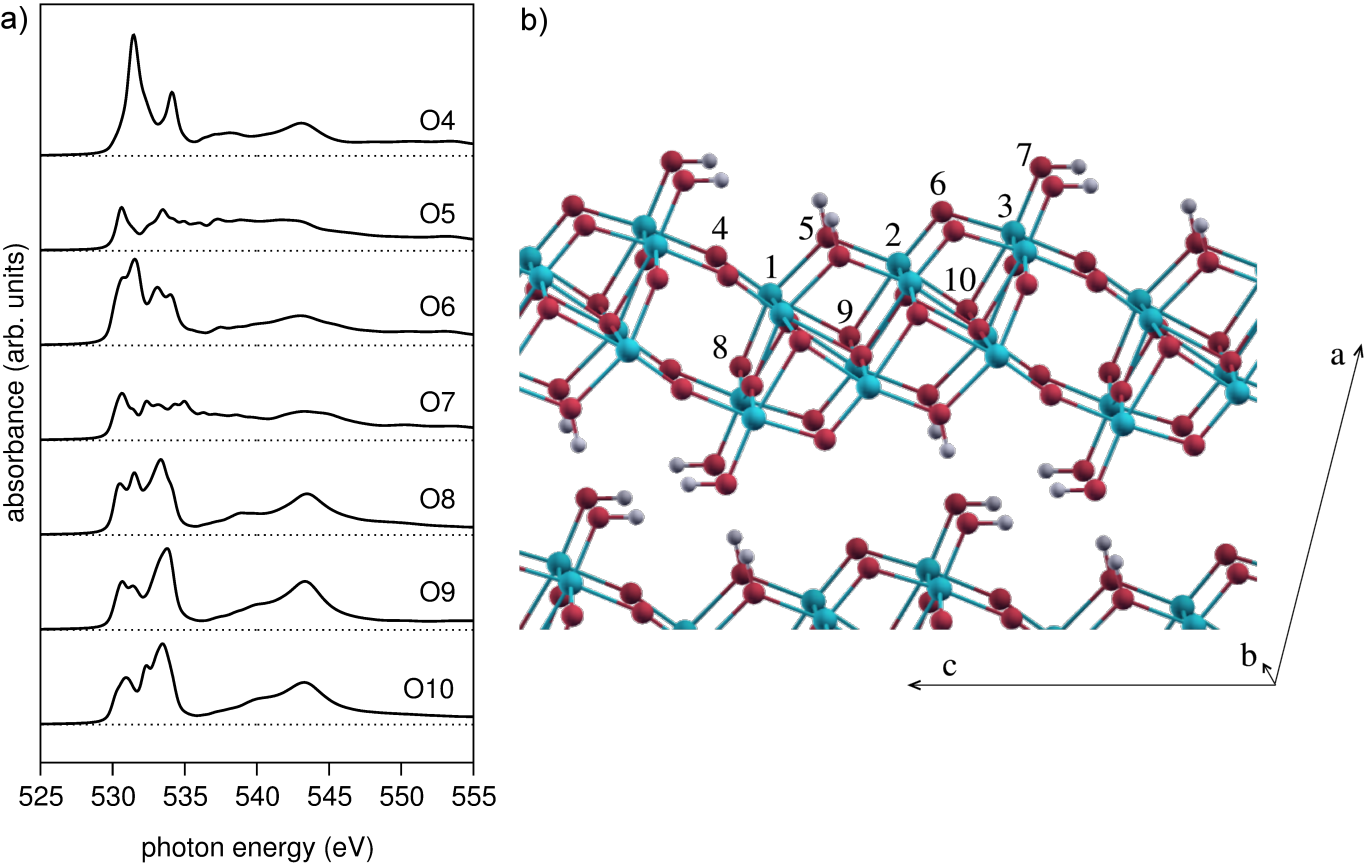
(a) Contribution to the O K-edge spectrum of oxygen atoms at different sites of the (Na,H)TiNTs structure (Figure 6b). (b) Ball-and-stick model of H_2_Ti_3_O_7_ [17], the generally accepted structure of (Na,H)TiNTs. Oxygen atoms in red with labels 4–10 for the seven nonequivalent sites, Ti atoms in blue are labeled 1–3 for the three Ti nonequivalent sites. Hydrogen atoms are in grey.

While all oxygen atoms are structurally equivalent in TiO_2_ anatase, there are seven different oxygen sites in the H_2_Ti_3_O_7_ structure and the measured O K-edge spectrum is an average over these seven sites. The individual spectra (Figure 6) are strongly different from each other, reflecting the different local environment of the O sites. All spectra except O5 and O7 show essentially the four main peaks A–D of anatase, albeit with extra fine structure in A and B, shifting of C and D, and considerable intensity variations. The spectra of the hydroxyl atoms O5 and O7 have a distinctly different shape where peaks A, B are strongly decreased and the dip between B and C is gone. This shows that the O K-edge spectra are highly sensitive to the number of H and Ti bonds, and thereby to the connectivity of the octahedra. In particular, for the non-hydroxyl O atoms, the A/B intensity ratio is found to be simply correlated with the O–Ti coordination: A > B for the 2-fold sites O4, O6; A ≈ B for the 3-fold site O8; and A < B for the 4-fold sites O9, O10. Thus, the A/B intensity ratio at the O K-edge XAS provides a direct local measure of the connectivity of the octahedra. This finding can be understood as follows: The O–Ti coordination of a given O atom equals its number of O-p–Ti-d sigma bonds. These bonds involve the Ti–e_g_ orbitals and, thus, correspond to peak B of the O K-edge spectrum. Therefore, the B-peak intensity increases (i.e., the A/B intensity ratio decreases) with increasing O–Ti coordination number, as seen in Figure 6.

Finally, the experimental observation that the (Na,H)TiNTs spectrum appears broader than that of anatase is easily understood as an averaging effect over several oxygen sites with markedly different bonding properties. Remaining small differences between the theoretical and experimental (Na,H)TiNT O K-edge spectrum may be attributed to the fact that the H_2_Ti_3_O_7_ model cannot fully account for the three-dimensional structure of the nanoscrolls. In the latter, the titanate sheets are bent and the stacking may slightly differ from the H_2_Ti_3_O_7_ crystal, which can be expected to influence the position of the hydroxyl groups [17].

Comparison of precise peak energy allows us to attribute specific peaks in the experimental O 1s spectrum to different oxygen locations within the crystal structure. The broader peak centred at 543 eV is primarily due to oxygen atoms O8, O9 and O10, i.e., oxygen in the layer centre. This is consistent with its similarity to peak D in anatase. The sharp peak at 531.5 eV, at the higher energy edge of peak A, is almost uniquely due to O4 and O6, i.e., oxygen atoms at the layer surfaces. Finally, the weak shoulder visible at 535 eV is seen only in the calculated spectra for O5 and O7, i.e., the surface hydroxyl groups. Thus the powerful combination of theoretical simulation with the high energy and spatial resolution of NEXAFS–TXM allows unambiguous identification of the presence of different oxygen bonding within individual layers of the (Na,H)TiNT.

In summary, we have shown that near-edge X-ray-absorption fine-structure spectroscopy combined with transmission X-ray microscopy (NEXAFS–TXM) is a powerful technique to investigate the electronic structure of titanate nanostructures when supported by multichannel multiple scattering and density functional theory. Already in one experimental data set several nanostructures are accessible to gain statistical results. By analysing the O K-edge and Ti L-edge of titanium-based materials (TiO_2_, SrTiO_3_, (Na,H)TiNTs) we show the effect of the connectivity of the TiO_6_ octahedra in the electronic states. (Na,H)TiNTs have an average connectivity between that of SrTiO_3_ and anatase, with differences in the splitting of the Ti L_3_–e_g_ line and O K-edge peaks ≈10 eV above threshold. These peaks correspond to the hybridization of O p with delocalized states and are thus particularly sensitive to structural changes around the O sites. This sensitivity allows direct comparison between experiment and simulations and subsequent assignment of specific peaks and shoulders in the O K-edge to oxygen in different bonding environments within individual titanate layers of the nanostructure. The excellent agreement between the experimental data and the theoretical modelling confirms the assignment of the (Na,H)TiNT structure to a layered titanate of the H_2_Ti*_n_*O_2_*_n_*_+1_ family. High-level spectroscopic modelling appears to be an important way to extend further the resolution of NEXAFS–TXM towards the atomic limit.

## Experimental

### Materials

Sodium titanate nanotubes ((Na,H)TiNTs) were synthesized from anatase TiO_2_ (Aldrich, –325 mesh), and 10 M NaOH(aq) under hydrothermal conditions at 135 °C. The anatase (Aldrich, –325 mesh), and SrTiO3 (Alfa Aesar) were used as standard

#### Characterization

The morphology of the synthesized material was investigated with a TEM (Jeol 2100, 200 keV).

The NEXAFS spectra were recorded with the TXM installed at the undulator beamline U41-XM at BESSY II, Berlin.

XPS (X-ray photoelectron spectroscopy) measurements were also performed in a VERSAPROBE PHI 5000, equipped with a monochromatic Al Kα X-ray source. The relative amount of sodium was evaluated to be 12% in accordance with EDS (11%) [FE-SEM (Carl Zeiss, Supra 35 LV)].

## Supporting Information

File 1 Broadening of the O K-edge spectra, comparison of the XPS Ti spectra recorded on the anatase and on the (Na,H)TiNTs, and experimental details.
