# Supplementary material for: Towards atomic resolution in sodium titanate nanotubes using near-edge X-ray-absorption fine-structure spectromicroscopy combined with multichannel multiple-scattering calculations
Source: Beilstein J Nanotechnol. 2012 Nov 23;3:789–97. doi: 10.3762/bjnano.3.88 (PMC3512128; doi:10.3762/bjnano.3.88)
Supplement: File 1 — Broadening of the O K-edge spectra, comparison of the XPS Ti spectra recorded on the anatase and on the (Na,H)TiNTs, and experimental details. [file Beilstein_J_Nanotechnol-03-789-s001.pdf]

Supporting Information
for

Towards atomic resolution in sodium titanate nanotubes using near-edge X-ray-absorption fine-structure spectromicroscopy combined with multichannel multiple-scattering calculations

Carla Bittencourt[*1], Peter Krüger[2], Maureen J. Lagos[3], Xiaoxing Ke[3], Gustaaf Van Tendeloo[3], Chris Ewels [4], Polona Umek[5,6] and Peter Guttmann[7]

Address: [1]ChIPS, University of Mons, B-7000, Mons, Belgium, [2] ICB, UMR 6303 CNRS-Université de Bourgogne, F-21078 Dijon, France, [3]EMAT, University of Antwerp, B-2020, Antwerp, Belgium, [4] Institut des Matériaux de Nantes (IMN), Université de Nantes, CNRS, Nantes, France, [5]Jožef Stefan Institute, Jamova cesta 39, 1000 Ljubljana, Slovenia, [6]Center of Excellence NAMASTE, Jamova cesta 39, 10000 Ljubljana, Slovenia and [7]Helmholtz-Zentrum Berlin für Materialien und Energie GmbH, Institute for Soft Matter and Functional Materials, Albert-Einstein-Str. 15, 12489 Berlin, Germany

Email: Carla Bettencourt* - carla.bittencourt@umons.ac.be
*Corresponding author

Broadening of the O K-edge spectra, comparison of the XPS Ti spectra recorded on the anatase and on the (Na,H)TiNTs, and experimental details.

Broadening of the O K-edge spectra

Following the prescription used in the MXAN code [1] the O-K edge spectra were broadened with a Lorentzian function of energy-dependent width $\Gamma(E)=\Gamma_c+A_s(0.5 + \arctan((E-E_s)/b)/\pi)$. Here Γ_c is the natural width of the O 1s hole (0.156 eV) and E_s is the step position, which was taken as the plasmon peak of anatase (12.7 eV) [2]. The parameters A_s = 1 eV (step height) and b = 2 eV have been adjusted to the experiment. The same parameter values were used for anatase and the (Na,H)TiNTs.

XPS study of the sample

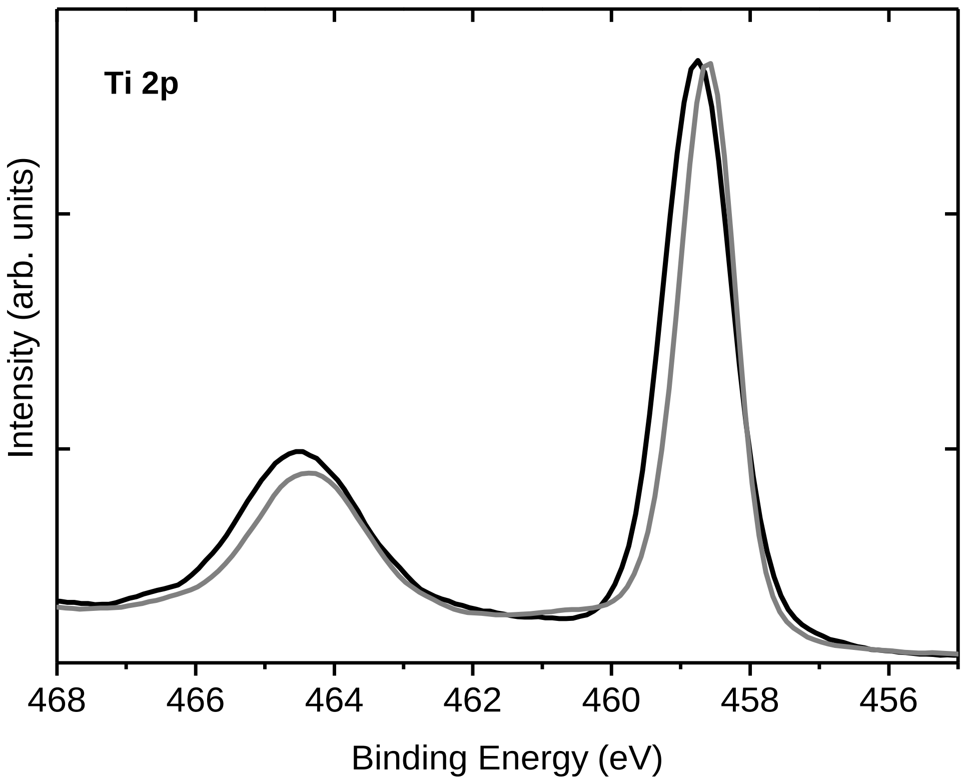

Figure S1: Comparison of the Ti 2p XPS core-level spectra recorded on (Na,H)TiNTs (grey line) and on anatase (black line) reference powder used as starting material.

Experimental

Materials Sodium titanate nanotubes ((Na,H)TiNTs) were synthesized from TiO_2 microparticles and NaOH(aq) under hydrothermal conditions at 135 °C. Detailed experimental procedure is described in [3]. Elemental composition (excluding hydrogen) determined with electron dispersive spectrometry (EDS) in combination with scanning electron microscopy (SEM) shows sodium content of 11.2 wt %, titanium 45.3 wt % and oxygen 43.5 wt %, respectively. Our XPS measurements show the samples to be about 10 wt % intercalated Na, in agreement with the EDS values.

Characterization The morphology of the synthesized material was investigated with a TEM (Jeol 2100 and a Tecnai G2 at 200 kV). For TEM and NEXAFS–TXM analysis the nanotubes were sonically dispersed in ethanol and a drop of the solution was deposited onto a lacey carbon film supported by a copper grid. Note that due to electron-beam damage effects on the (Na,H)TiNTs, different grids were used for the electron microscopy and for the NEXAFS–TXM analysis.

The Ti 2p L-edge spectra were recorded with the TXM installed at the undulator beamline U41-XM at BESSY II, Berlin [4-6]. The setup of the instrument is analogous to a bright-field light microscope. A focusing spherical grating monochromator (FSGM) at the undulator beamline U41-XM at the electron storage ring BESSY II of the HZB delivers X-rays with photon energy E and the required high spectral resolution of $E/\Delta E \geq 4500$. A single reflection ellipsoidal shaped mirror condenser [7,4] is used to illuminate an object field of 15–20 μm.

In the best case the HZB TXM provides a high spatial resolution close to 10 nm (half-pitch) [8] and a spectral resolution up to $E/\Delta E \approx 10^4$. It allows measurements to be taken at room or liquid nitrogen temperature in a vacuum of 10^{-7} TORR. The spectra were recorded at room temperature in transmission mode by taking a sequence of images over a range of photon energies covering the investigated absorption edges with $E/\Delta E \geq 4500$. Note that the exit slit of the monochromator was set to 5 μm, which corresponds to a calculated spectral resolution of $E/\Delta E = 2 \times 10^4$. For the present study a zone plate objective with an outermost zone width of 40 nm was used to image the sample onto a cooled back-illuminated soft X-ray CCD camera (Roper Scientific, PI SX1300).

The NEXAFS spectra were normalized since the photon flux varies as a function of photon energy ($h\nu$) and time in the object field (x, y). The normalization was performed by dividing the function $I(x, y, h\nu)$ recorded on the sample by the photon flux curve $I_o(x + \Delta x, y + \Delta y, h\nu)$

recorded in its sample free proximity at position ($x + \Delta x$, $y + \Delta y$). Both $I(x, y, h\nu)$ and $I_o(x, y, h\nu)$ were recorded in the same image stack since near each studied nanostructure bare regions permit the measurement of I_o.

The elemental compositions of the sample was investigated with a FE-SEM (Carl Zeiss, Supra 35 LV) equipped with an EDS (energy-dispersive X-ray spectrometer) element analysis system.

XPS (X-ray photoelectron spectroscopy) measurements were also performed to determine chemical composition, in a VERSAPROBE PHI 5000 from Physical Electronics, equipped with a monochromatic Al Kα X-ray source with a highly focused beam size that can be set from 10 to 300 µm. The energy resolution was 0.6 eV. For the compensation of the built up charge on the sample surface during the measurements, a dual-beam charge neutralization composed of an electron gun (~1 eV) and argon ion gun (≤10 eV) was used. The relative amount of sodium was evaluated to be 12% in accordance with EDS (11%).

References

1. Benfatto, M.; Della Longa, S.; D'Angelo, P. *Phys. Scr.* **2005,** *T115,* 28–30. doi:10.1238/Physica.Topical.115a00028
2. Mo, S.-D.; Ching, W. Y. *Phys. Rev. B* **1995,** *51,* 13023–13032. doi:10.1103/PhysRevB.51.13023
3. Umek, P.; Cevc, P.; Jesih, A.; Gloter, A.; Ewels, C. P.; Arcon, D. *Chem. Mater.* **2005,** *17,* 5945–5950. doi:10.1021/cm050928w
4. Guttmann, P.; Zeng, X.; Feser, M.; Heim, S.; Yun, W.; Schneider, G. *J. Phys.: Conf. Ser.* **2009,** 012064–012066.
5. Schneider, G.; Guttmann, P.; Heim, S.; Rehbein, S.; Mueller, F.; Nagashima, K.; Heymann, J. B.; Muller, W. G.; McNally, J. G. *Nat. Methods* **2010,** *7,* 985–987. doi:10.1038/nmeth.1533
6. Guttmann, P.; Bittencourt, C.; Rehbein, S.; Umek, P.; Ke, X.; Van Tendeloo, G.; Ewels, C. P.; Schneider, G. *Nat Photon* **2012,** 6, 25-29.
7. Zeng, X.; Duewer, F.; Feser, M.; Huang, C.; Lyon, A.; Tkachuk, A.; Yun, W. *Appl. Opt.* **2008,** *47,* 2376–2381. doi:10.1364/AO.47.002376
8. Rehbein, S.; Guttmann, P.; Werner, S.; Schneider, G. *Opt. Express* **2012,** 20, 5830-5839. doi:10.1364/OE.20.005830
